# Assistance and health care provided to adolescents with chronic and immunosuppressive conditions in a tertiary university hospital during the COVID-19 pandemic

**DOI:** 10.6061/clinics/2021/e2688

**Published:** 2021-03-15

**Authors:** Sofia S.M. Lavorato, Alberto C. Helito, Vera P.M.F.R. Barros, Deborah F.P. Roz, Ligia P. Saccani, Lorena V.M. Martiniano, Lívia M.L. Lima, Dandara C.C. Lima, Benito Lourenço, Rosa M.R. Pereira, Bruno Gualano, Clovis A. Silva, Ligia B. Queiroz

**Affiliations:** I Instituto da Crianca e do Adolescente, Hospital das Clinicas HCFMUSP, Faculdade de Medicina, Universidade de Sao Paulo, Sao Paulo, SP, BR.; II Divisao de Reumatologia, Hospital das Clinicas HCFMUSP, Faculdade de Medicina, Universidade de Sao Paulo, Sao Paulo, SP, BR.; III Grupo de Pesquisa em Fisiologia Aplicada e Nutricao, Escola de Educacao Fisica e Esporte, Faculdade de Medicina FMUSP, Universidade de Sao Paulo, Sao Paulo, SP, BR.; IV Centro de Pesquisa em Alimentos, Universidade de Sao Paulo, Sao Paulo, SP, BR.

The coronavirus disease (COVID-19) pandemic has led to social distancing and isolation measures worldwide (1,2). Quarantine began on July 20, 2020 in São Paulo State, Brazil (3). Institutional guidelines of the Hospital das Clínicas of FMUSP (HCFMUSP) released on March 18, 2020 recommended postponing medical and multidisciplinary appointments, outpatient procedures, and elective surgeries. As a result, the regular follow-up of a considerably large group of adolescents living with pre-existing chronic conditions was provisionally scheduled at longer intervals or even paused.

Telemedicine and telepsychology were used during the quarantine period. These models have become a challenge in clinical practice because they are not typically used in the care of pediatric patients with chronic conditions (4).

The most prevalent conditions observed in the adolescents seen in our institution are asthma, obesity, cancer, transplantation, chronic kidney disease, and autoimmune diseases (5,6). These pediatric patients often show severe and complex conditions with an unpredictable course that require several medications and the input from multiple pediatric specialists. Despite the concern that these patients may have severe infections related to other viral agents, such as adenovirus, rhinovirus, influenza, and respiratory syncytial virus, knowledge of the overall impact of COVID-19 in this population remains incomplete (7-9).

Adolescence is a phase of relevant biological, social, and affective changes. Teenagers gradually become more independent, strengthen relationships with friends, and start romantic and sexual partnerships. These aspects have changed in the face of the social distancing experience (10,11).

For adolescents with chronic diseases from the Instituto da Criança e do Adolescente of HCFMUSP, the fear of being infected by severe acute respiratory syndrome coronavirus 2 (SARS-CoV-2) and becoming seriously ill is easy to understand. Most of them are aware of the immunosuppression caused by their diseases or treatments they receive. Therefore, the interruption of regular appointments may lead to concerns regarding clinical worsening (4,6).

In response, a quantitative and qualitative multidisciplinary and multi-professional research project was developed to evaluate the pandemic’s impact on the physical and mental health of the chronically ill adolescents who are followed at our university hospital. The study was registered at ClinicalTrials.gov (31314220.5.0000.0068) and approved by the Brazilian National Committee for Research Ethics (CONEP number 4.081.961).

The methodology included the construction of semi-structured forms and use of instruments for assessing health-related quality of life and disease activity parameters for adolescents with several pre-existing chronic conditions, such as gastrointestinal, liver, rheumatological, and nephrological diseases, obesity, and pediatric liver and kidney transplant.

The choice for these specific groups was because of the fact that they share similar clinical characteristics and therapies. Indeed, these groups were at risk of disease flares or additional comorbidities during this pandemic. They could also be at higher risk of contracting COVID-19 since they were subjected to immunosuppressive and immune-modifying drug treatments, dialysis, or liver/kidney transplantations.

This survey tool was sent electronically by e-mail or texts to patients and their relatives. The invitation to participate in the research was made via an initial phone call from a physician who was part of the patient’s support team.

Early contact via phone calls played a pivotal role in the qualitative methodology design. The postponing of appointments hindered health follow-up, making this individual call an opportunity to address possible health care needs of each patient. At that moment, an important communication channel was created between adolescents, their families, and their health care providers. As such, any patient’s needs, questions, and requests were acknowledged by the multidisciplinary health team at the start of the study.

Researchers met weekly to exchange experiences and to refer the adolescents to the specific health staff responsible for addressing any issues, such as psychological demands, treatment adjustments, and questions regarding exams or appointment schedules. Furthermore, the multidisciplinary teams involved in the research were able to deepen their understanding of their patients.

This study included several units, divisions, and departments of the University of São Paulo. Several pediatric specialties, such as physical education, nutrition, psychology, and orthopedics, were involved in the project. This disciplinary plurality allowed an special opportunity for mutual support and exchange of experiences among health teams facing patients and families’ needs, resulting in a transdisciplinary health care action.

After the first phone contact, the survey was sent via the RedCap system. Patients were assisted in answering the survey using computers, tablets, or smartphones.

Patients were also invited to participate in a home-based exercise training program supervised by the physical education team. The nutrition team contacted the adolescents to invite them to fulfill food recalls for three non-consecutive days. Throughout the study, the physicians who had made the first contact with the adolescents continued to follow them by phone and/or text messages.

Overall, 355 adolescents with pre-existing chronic and immunosuppressive conditions were evaluated. Most reported an increase in screen time and sleep after midnight, and they also mentioned a decrease in their familýs financial status during the pandemic.

The psychology team created a guide for the physicians to follow during the call that included four questions to be answered by the patients or their relatives. For those who suggested special psychological needs, the psychology team developed a delimited online appointment proposal. In fact, the teenagers could request psychological support at any moment, such as during physical training sessions, nutritional inquiries, or in response to the text messages sent by the physicians. The patients’ relatives could also ask questions regarding treatment or request psychological support for the teenagers. Some adolescents complained about the worsening of pre-existing symptoms during the pandemic, such as generalized anxiety, sadness, depression, a lack of energy, and even suicidal thoughts. These same symptoms were also reported by some patients who had never complained about them before the pandemic.

The psychology team assisted 22 adolescents with mental health issues, 70% of whom were 13-15 years of age. These patients were predominantly followed up by our hospital’s Nephrology Unit (37%). A significant number of patients reported emotional state changes and losses of concentration and social interaction as well as a lack of routine because of isolation. Reports also included sadness, anxiety, aggression, appetite change, depression, self-harming behavior, and suicidal thoughts. These reports have been observed in other adolescent cohorts assessing the psychological repercussions of quarantine because of the COVID-19 pandemic (12,13).

We also aimed to identify changes in chronic disease treatment during the pandemic. There were no cases of chronic disease flares or worsening, something that the pediatric specialists were deeply concerned about during the pandemic (14,15). Fears related to SARS-CoV-2 infection were also evaluated. Previous psychological/psychiatric treatments were tracked to understand how isolation affects the mental health of psychologically vulnerable patients (16).

This study enabled an ethical and committed link between adolescent patients, their families, caregivers, and health care providers, making it possible to constantly assist patients during the pandemic. It can be argued that this emerging model of health care grounded in research was successful at providing patients health support and, at the same time, produced novel data that can better inform the treatment of this population during such as unprecedent crisis. Certainly, the findings, experiences, and failures from this project will help clinicians better manage adolescents with chronic diseases during the COVID-19 pandemic.
